# GaAs-Based Superluminescent Light-Emitting Diodes with 290-nm Emission Bandwidth by Using Hybrid Quantum Well/Quantum Dot Structures

**DOI:** 10.1186/s11671-015-1049-2

**Published:** 2015-08-25

**Authors:** Siming Chen, Wei Li, Ziyang Zhang, David Childs, Kejia Zhou, Jonathan Orchard, Ken Kennedy, Maxime Hugues, Edmund Clarke, Ian Ross, Osamu Wada, Richard Hogg

**Affiliations:** Department of Electronic and Electrical Engineering, University of Sheffield, North Campus, Broad Lane, Sheffield, S3 7HQ UK; Department of Electronic and Electrical Engineering, Centre for Nanoscience and Technology, EPSRC National Centre for III-V Technology, University of Sheffield, Broad Lane, Sheffield, S3 7HQ UK; Center for Collaborative Research and Technology, Kobe University, Kobe, 657-0013 Japan

**Keywords:** Superluminescent Light-Emitting Diode, Quantum well, Quantum dot

## Abstract

A high-performance superluminescent light-emitting diode (SLD) based upon a hybrid quantum well (QW)/quantum dot (QD) active element is reported and is assessed with regard to the resolution obtainable in an optical coherence tomography system. We report on the appearance of strong emission from higher order optical transition from the QW in a hybrid QW/QD structure. This additional emission broadening method contributes significantly to obtaining a 3-dB linewidth of 290 nm centered at 1200 nm, with 2.4 mW at room temperature.

## Background

Optical coherence tomography (OCT) is a non-invasive biomedical imaging technique that can form 3D images of the near-surface (1–2 mm deep) of biological tissue [1]. The axial resolution of the imaging system is governed by the coherence length of the light source [2], prompting a drive for the development of light sources with broad spectral emission. GaAs-based quantum dot materials have attracted significant attention due to the achievable emission wavelengths matching the minimum in scattering of skin tissue [3]. Furthermore, quantum dot (QD) materials exhibit inhomogeneous broadening, as the self-assembled QDs are not identical in shape, size, and composition [4], and it is possible to harness the strong state-filling effects [5]. A suitable light-emitting device for OCT applications is the superluminescent diode (SLD) which combines many aspects of the laser diode but requires a range of techniques to be employed in order to inhibit lasing and ensure a broad emission bandwidth [6]. Quantum dot-based SLDs [7–10] have been applied to OCT of skin tissue [11] using a QD SLD with 85-nm spectral bandwidth centered at 1250 nm, which resulted in an ~7-μm axial resolution of the OCT system [12].

In order to further enhance the system resolution, ever broader spectral bandwidth sources are required. We previously proposed and demonstrated a hybrid active element incorporating both InGaAs quantum wells (QWs) and QDs [13]. The QW was spectrally positioned to be resonant with the second excited state of the QD ensemble, and we previously showed that the spatial position of the QW within the multi-layer stack of the active element was critical [14]. Such structures exhibited an ~300-nm-wide modal gain spanning 1100–1400 nm [13] and exhibited simultaneous three-state lasing [15].

In this letter, we report on a newly observed feature in the broadening of the emission spectrum of hybrid QW/QD active elements and the strain/piezoelectric modulation of the QW by the QD layers. We go on to demonstrate the SLD based upon the hybrid QW/QD active element. We focus attention firstly on the spatial strain/piezoelectric modulation of the QW due to the QD layers. We experimentally demonstrate that in the hybrid QW/QD structure, this effect produces both broader QW transitions and more pronounced high-order transitions, both of which can be expected to be beneficial for broad spectral bandwidth devices. Based on this newly observed effect, we then report on the first hybrid QW/QD SLD, which is capable of emitting an emission bandwidth of 290 nm centered at 1200 nm with a corresponding output power of 2.4 mW at room temperature. This is achieved due to the combined effects of introducing a single QW into a multi-stack of QD layers, spectrally positioned to offset the loss due to the second excited state (ES2) of the QDs, as well as introducing the high-order QW transitions due to the QD strain-induced piezoelectric potential [16] and/or directly through strain effects [17]. The hybrid QW/QD SLD is then assessed for application in an OCT system, and the analysis of the emission spectrum indicates that this device should enable a predicted axial resolution of ~2.9 μm, a factor of 3–5 times better than the standard OCT system using conventional SLDs [18], indicating that hybrid QW/QD SLDs are highly desirable for submicrometer-resolution OCT imaging.

## Methods

### Epitaxy Growth and Device Fabrication Details

To investigate the effect of QD-induced strain on QW modulation, three p-i-n LED test structures named “QW-only,” “QW + QD,” and “QD-only” were studied. These LED test structures were grown by a V90 solid source molecular beam epitaxy (MBE) reactor on a (100) Si-doped substrate. The epitaxial sequence for the three test structures is essentially identical except for the growth of the active region. The epitaxy for the three test structures all starts with a 200-nm-thick n-type GaAs buffer layer (2 × 10^18^ cm^−3^) followed by a 400-nm n-type (5 × 10^17^ cm^−3^) Al_0.35_Ga_0.65_As lower cladding layer, and above this is an undoped active region, followed by a 400-nm p-type (5 × 10^17^ cm^−3^) Al_0.35_Ga_0.65_As upper cladding layer and finally a 100-nm highly p-doped (1 × 10^19^ cm^−3^) GaAs contact layer. For the growth of the active region, for the QW-only test sample, a 7-nm undoped In_0.3_Ga_0.7_As QW is sandwiched between 96.5 nm of undoped GaAs. For the QW + QD test sample, firstly, 56.5 nm of undoped GaAs and then a 7-nm undoped In_0.3_Ga_0.7_As QW were grown; this was followed by the deposition of 35 nm of undoped GaAs and then one undoped In_0.18_Ga_0.82_As/InAs/In_0.18_Ga_0.82_As dot-in-well (DWELL) layer [19, 20]. Due to the comparatively thick barrier between the QW and QD, no electric coupling between QW and QD layers is expected [21]. The growth of the active region for the QD-only test sample is nominally identical to that for the QW-only test sample, but with the QW being replaced by a single DWELL layer identical to that used in the QW + QD test structure.

A hybrid QW/QD laser structure consisting of a single In_0.34_Ga_0.66_As QW and six-stack InAs DWELL layers embedded in an AlGaAs waveguide was also grown on a (100) Si-doped GaAs substrate in a V90 MBE reactor. A detailed description of this structure can be found in ref. [13]. The hybrid QW/QD SLDs were fabricated in a tilted-waveguide structure following standard ridge laser processing. Wire ridges of 5 and 10 μm were defined by inductively coupled plasma etching to a depth of 1.5 μm. The SLDs described in this letter were 5 μm in width and 2 mm in length. The stripe window was tilted at 7° to inhibit lasing. No facet coating was applied. To characterize the spontaneous emission and photocurrent (PC) spectroscopy, standard optical access mesa diodes were also fabricated [14]. PC was carried out using a tungsten halogen lamp, dispersed by a Bentham M300 single monochromator. The PC signal was recorded using standard lock-in techniques.

### Strain Modulation of the QW by the QD Layers

**Fig. 1:**
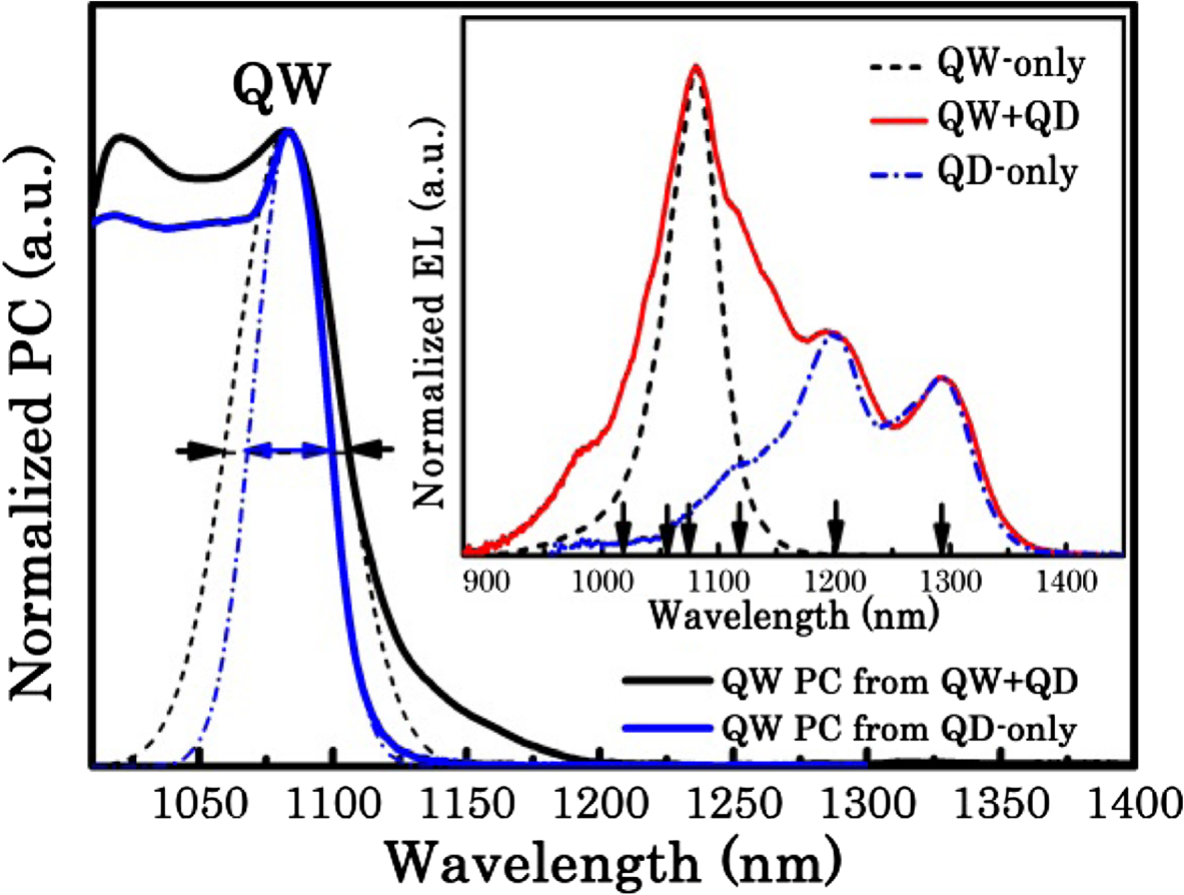
Normalized QW PC spectra for the QW-only sample and QW + QD sample at an electric field of 8 kV cm^−1^ at room temperature. The *inset* shows the normalized EL spectra obtained from mesa diodes (200 μm) for the QW-only, QW + QD, and QD-only samples at room temperature. The marks at the *bottom* (from *right* to *left*) indicate the following transitions: QD GS, QD ES1, QD ES2, QW_e1-hh1_, QW_e1-hh2_, and QW_e1-hh3_

Figure 1 shows the normalized QW PC spectra for the QW-only test sample and the QW + QD test sample at an electric field of 8 kV cm^−1^ (which is a low reverse bias, but where the PC from the QD ground state (GS) reaches a local maximum indicating that tunneling from the QD GS is maximized) at room temperature. The QW PC signal of the QW + QD test sample is obtained by subtracting the normalized QD PC from the QW + QD PC. For the QW-only test sample, the PC spectrum shows a clear, sharp peak at 1083 nm, which indicates the lowest energy transition of the QW. Very good agreement between the lowest energy transitions of the QW in the QW-only and QW + QD test samples is found. However, regarding the measured linewidth (which is the full width at half maximum of the line that corresponds to the best fit assuming the sum of Gaussian functions), the QW PC from the QW + QD test sample is found to have a broader linewidth than that from the QW-only test sample. The increased linewidths (from 30 to 47 nm), along with considerable band tailing (1150–1200 nm), are attributed to the modulation of the QW by a spatial strain modulation induced by the highly strained QDs [17, 22–24], or due to the formation of a piezoelectric potential due to the strained QDs [16].

The inset in Fig. 1 shows the normalized electron luminescence (EL) spectra obtained from mesa diodes for QW-only, QW + QD, and QD-only test samples at room temperature. The QW-only and QW + QD test samples are driven with the same current density of 300 A cm^−2^. In order to investigate the contribution of the QDs to the emission in the QW + QD test sample, the QD-only test sample is injected at a specific current density (160 A cm^−2^) where the ratio of the emission from the QD ground state and excited state is the same as that of the QW + QD test sample. It is assumed that at these current densities, the QD carrier occupancy in the QW + QD test sample and QD-only test sample is the same. As indicated in the inset in Fig. 1, the EL spectrum for the QD-only test sample shows a series of well-defined features at 1293, 1200, and 1116 nm, which are attributed to the ground state, the first excited state, and the second excited state of the QD transitions. For the QW-only test sample, only a single peak at 1084 nm is observed attributed to the lowest energy transition of the QW (e1-hh1). By contrast, for the QW + QD test sample in addition to the e1-hh1 transition, two more features at 1058 and 1018 nm are observed. The energies of these features are in agreement with the predicted energies of higher order QW transitions (e1-hh2 and e1-hh3) from modeling of a single QW [25].

These higher order QW transitions observed in the QW + QD test sample are highly desirable for achieving a broad bandwidth [26]. We note that for the photocurrent, features are also observed at these wavelengths. These higher order transitions should have a very low oscillator strength in a regular QW, and their appearance with significant (i.e., non-zero) PC and EL is attributed to the strain/piezoelectric modulation of the QW by the QDs. We note that the emission from the EL spectrum is significantly greater than the absorption observed in the PC spectra, which suggests that piezoelectric modulation is dominating in this case, though further structural and spectroscopic measurements are required to confirm this. To summarize the results obtained from these test samples, QD strain modulation of the underlying QW, which is deposited *before* the QDs, through either direct strain or indirect piezoelectric modulation, results in a broadened linewidth of the e1-hh1 transition and leads to larger oscillator strengths for the higher order QW transitions (e1-hh2, e1-hh3). Based on this observation, the higher order QW transitions may be expected to play a significant role in further enhancing the spectral bandwidth of a hybrid QW/QD SLD. We therefore moved to exploit higher order QW emission in a multi-layer QD structure which contains a single QW spatially positioned at the n-side of the stack [14] and spectrally positioned so as to have the e1-hh1 QW transition spectrally overlapping with the second excited state of the QD ensemble.

**Fig. 2:**
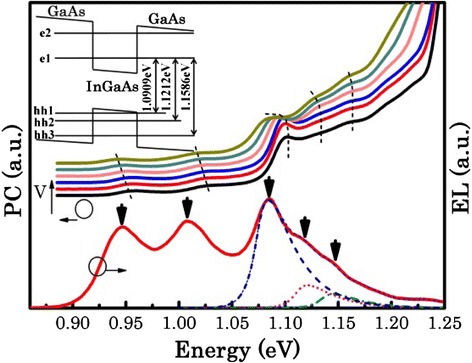
PC spectra as a function of applied reverse bias and an EL spectrum at a current density of 1.4 kA cm^−2^ for hybrid QW/QD samples measured normal to the surface of an optical access mesa diode at room temperature. The *inset* shows the modeling results of the band structure for a single InGaAs quantum well. The QW energy levels were calculated using an effective mass approximation method. This model included temperature- and strain-dependent effects and standard material parameters. A single 7-nm 34 % InGaAs QW was simulated clad with GaAs barriers on either side. An electric field of 10 kV cm^−1^ was also applied to the simulation structure

Figure 2 shows the PC spectra as a function of applied reverse bias and an EL spectrum at a current density of 1.4 kA cm^−2^ for the hybrid QW/QD laser sample, measured normal to the surface of an optical access mesa diode at room temperature. In the PC spectra, a linear shift of the QD interband transitions (GS and first excited state (ES1)) to lower energy is clearly observed, as expected for quantum dots with a permanent dipole moment at 0 V [27]. A strong PC signal is observed at ~1.09 eV. Comparison to data for a control sample (not shown) that contains only QDs indicates that this peak is due to the QW. As the applied reverse bias is increased, a quadratic shift of this transition to lower energy and a concomitant broadening of the PC peak are observed in agreement with the quantum-confined Stark effect (QCSE) for a QW [28]. The agreement with the predicted transition (see inset) and QCSE allows us to attribute this feature to absorption by the e1-hh1 QW transition. At low reverse bias, weak transitions to higher energy at ~1.12 and ~1.15 eV are also observed, which we attribute to the QW transitions due to e1-hh2 and e1-hh3, due to the agreement with predicted QW transition energies as shown in the inset in Fig. 2 and our expectation that the QD strain modulation of the QWs will enhance these transitions. At high reverse bias, transitions for e1-hh2 and e1-hh3 are also observed. Compared to the e1-hh1 transition, a relatively small energy shift with bias is seen for e1-hh2 and e1-hh3 in agreement with the higher energy transitions being less sensitive to the applied reverse bias than lower energy transitions, and is in agreement with modeling.

The EL spectrum (Fig. 2 lower) shows clear luminescence peaks at 0.95 and 1.02 eV, due to the GS and ES1 transitions of the ensemble of the InAs QDs. The peak which dominates the spectrum at ~1.09 eV has been shown to be due to the lowest energy transition of the single In_0.34_Ga_0.66_As QW as opposed to higher order QD transitions [13–15]. Two more features are observed at ~1.12 and ~1.15 eV. Very good agreement between the QD and QW transition energies in PC and EL is observed. We therefore attribute the features observed at ~1.12 and ~1.15 eV in EL to the transitions involving the lowest electron state and higher order hole states in the QW (e.g., e1-hh2, e1-hh3). In order to investigate the contribution of the high-order QW transitions (0e1-hh2, e1-hh3) to the spectral bandwidth of the device, we carried out line-shape fitting to the spontaneous emission spectrum as shown in Fig. 2. The technique used for modeling the emission is described in [29]. The higher order transitions were found to be contributing ~33 % of the power to the emission spectrum at this current density. This significant enhancement to the shorter wavelength side of the emission band is very attractive for our application, but the origin is not entirely clear at present. Deformation of the crystal (direct strain modulation of the underlying QW layer) may be responsible, but the separation of the QW and QD layers is rather large, at 45 nm. Considering the low absorption cross section, yet strong emission from these forbidden transitions, a QD-induced piezoelectric potential within the QW layer [16] may be the most likely explanation. This highlights the need for further structural and optical characterization and simulation work to determine the relative strengths of these two effects.

## Results and Discussion

**Fig. 3:**
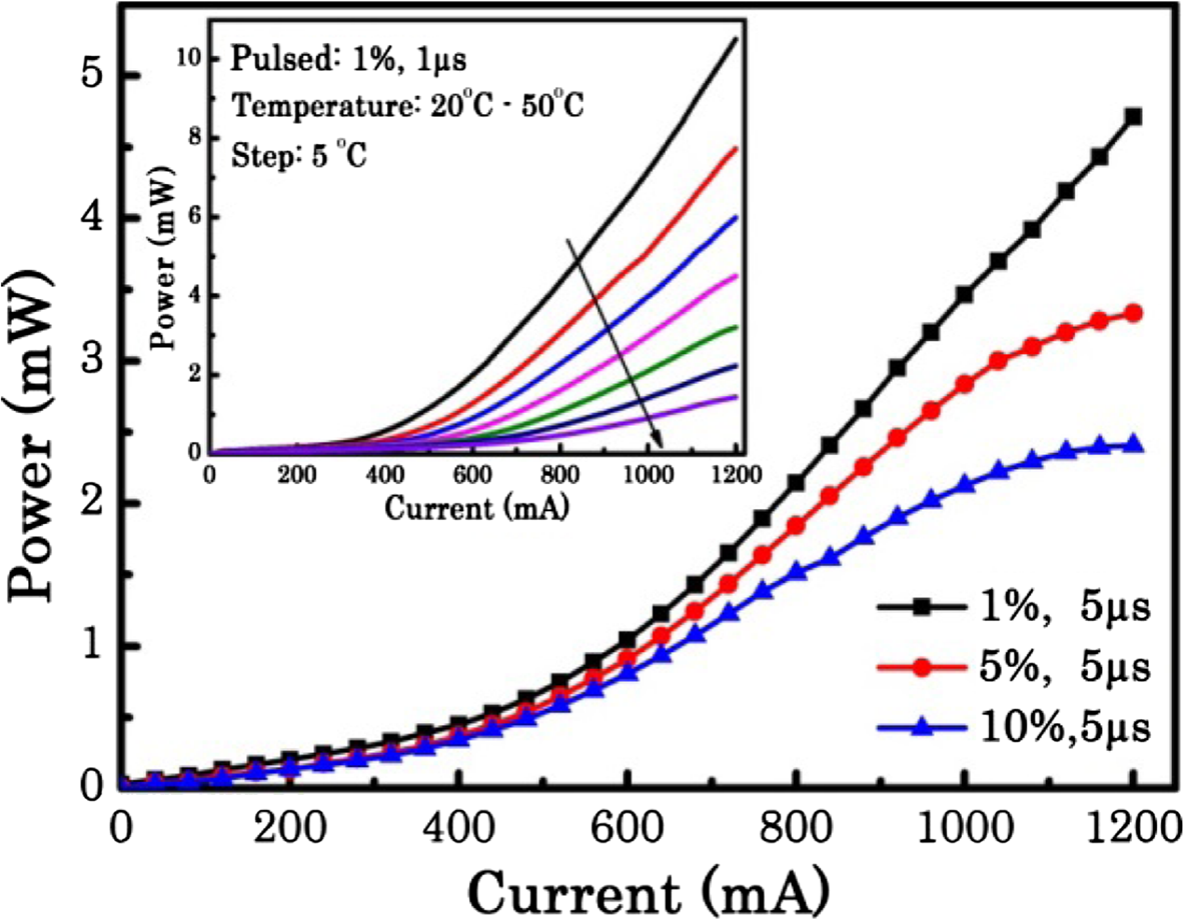
L-I curve of the hybrid QW/QD SLD as a function of duty cycle. The *inset* shows the output power as a function of temperature at fixed duty cycle and pulse width (1 %, 1 μs)

Figure 3 shows the output power as a function of the injection current (light-current-voltage (L-I)) of the hybrid QW/QD SLD. Measurements were taken with the device mounted epi-side up with a sub-mount temperature of 20 °C with no active cooling under pulsed operation. Under pulse conditions of 1 % duty cycle and 5-μs pulse width, superluminescent behavior is evidenced by the superliner increase in optical power with increasing current. A power of 4.6 mW was measured at 1200 mA, and it should be noted that this is an underestimation of the power as a wavelength of 1300 nm (0.95 eV) was assumed in calculating the power. The inset in Fig. 3 plots the output power as a function of temperature at a fixed duty cycle and pulse width (1 %, 1 μs). At 20 °C, a maximum output power of 11 mW is achieved. By increasing the temperature from 20 to 50 °C, the output power decreases significantly. This strong effect of temperature on the L-I characteristics is attributed to increased non-radiative recombination and carrier escape from the heterostructures with increasing temperature [30]. As seen in Fig. 3, increasing the duty cycle from 1 to 10 % reduced the maximum output power from 4.6 to 2.4 mW. Comparison to the inset indicates that self-heating of the device due to non-ideal heat sinking leads to a 10–15 °C temperature increase of the junction at 1200 mA when we move from 1 to 10 % duty cycle. These thermal effects may be reduced in the future by either p-doping the active region [31] or using the epi-down mounting process [32].

**Fig. 4:**
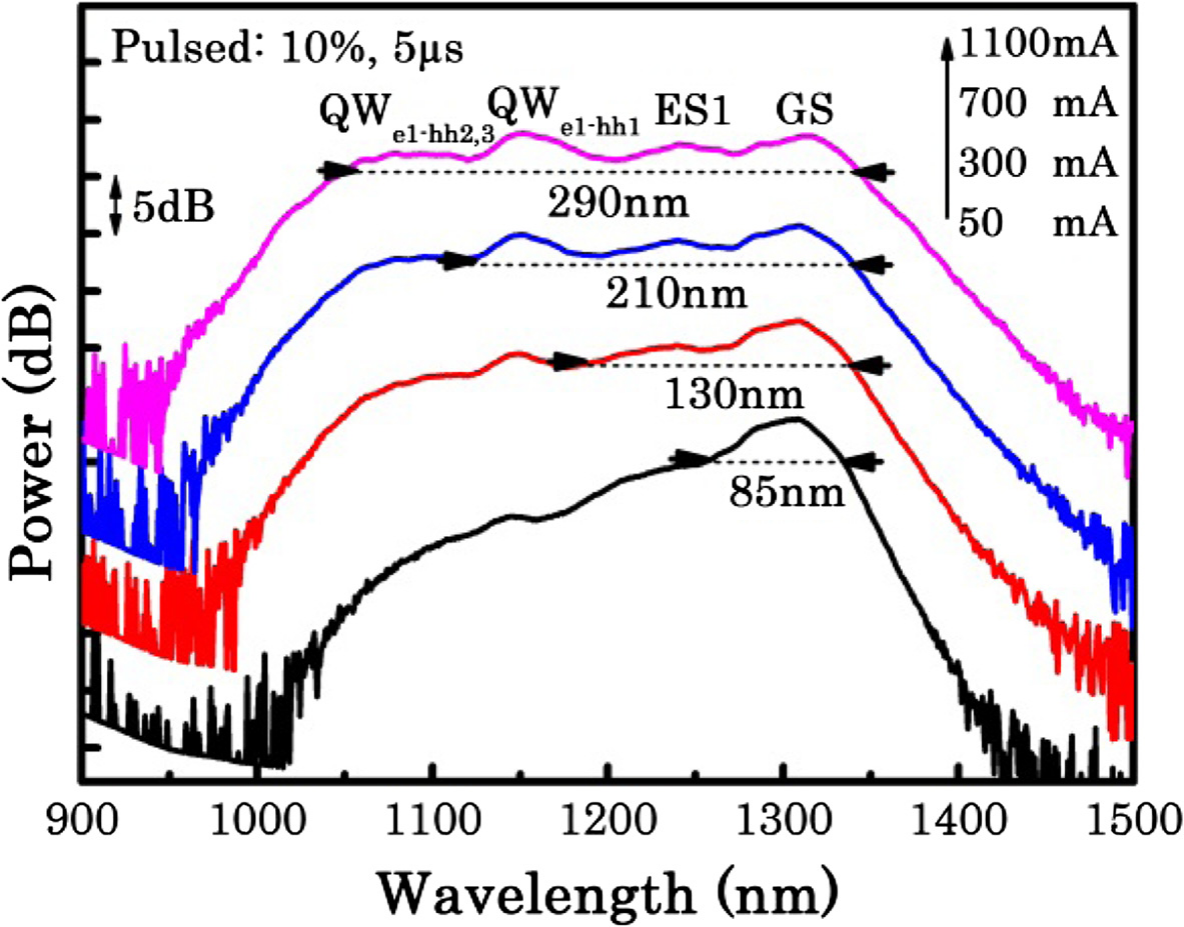
Output power spectrum as a function of the injection current under pulsed operation (10 % duty cycle and 5-μs pulse width) measured at room temperature

Figure 4 plots the output power spectrum as a function of the injection current under pulsed operation (10 % duty cycle and 5-μs pulse width) measured at room temperature. The spectra are offset for clarity. At a drive current of 50 mA, the emission is dominated by the ground state of the QDs at 1305 nm. As the injection current is increased to 200 mA, the ground state peak intensity saturates and the carriers begin to fill ES1 at 1230 nm. With the further increase of the drive current over 500 mA, the QD emission begins to saturate, while an emission peak at 1150 nm due to the lowest energy transition of the QW increases and gradually dominates the emission spectrum at the highest current. This is in line with our previous work where we measured the gain spectrum of this material and have observed that the modal gain for GS and ES1 of the QDs is clamped at these current densities, while the QW increases in modal gain with further increasing current density [13]. In addition, at higher current, a shoulder in the emission spectrum between 1050 and 1100 nm emerges, which further contributes to the full width at half maximum (FWHM) of the emission spectrum. These spectral features between 1050 and 1100 nm were previously attributed to higher order transitions of the QW as discussed in Fig. 2.

**Fig. 5:**
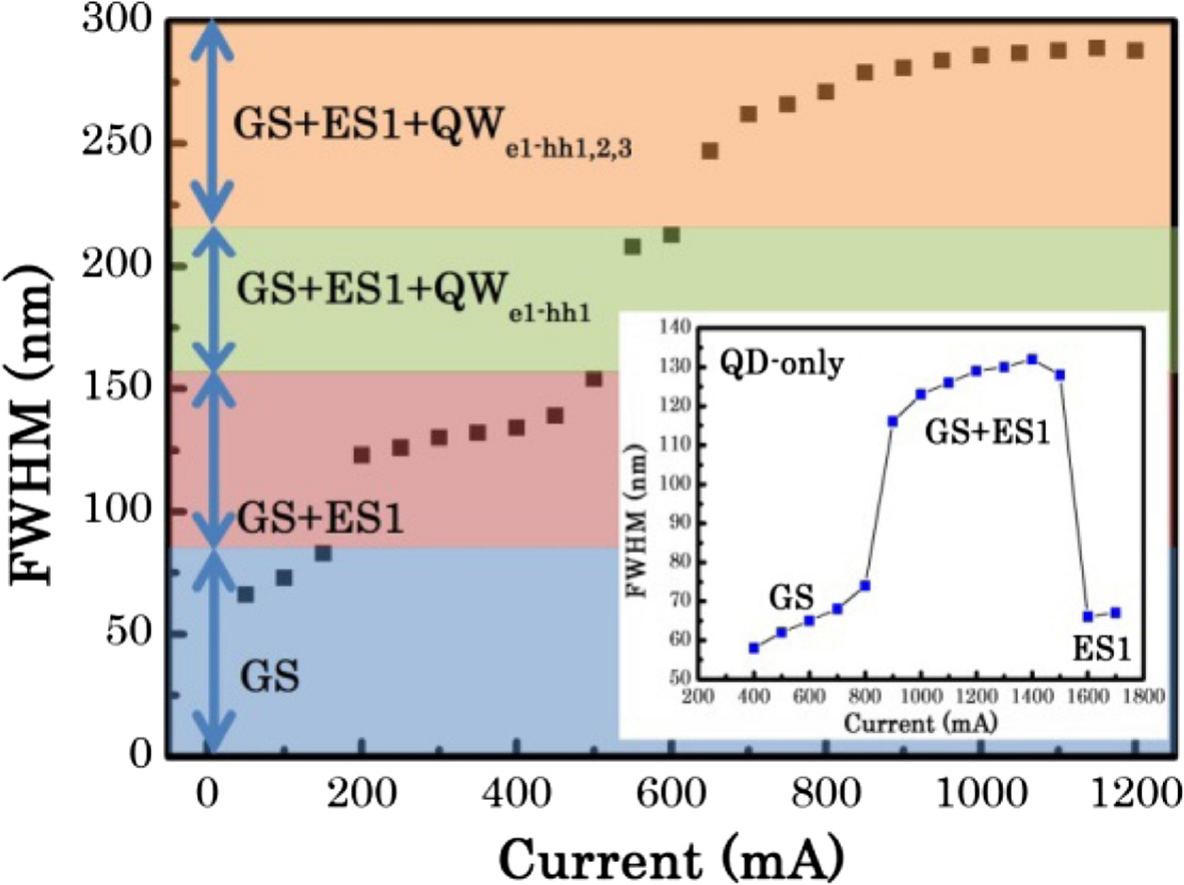
Evolution of the FWHM versus the injection current obtained from the output power spectrum of the hybrid QW/QD SLD. The *inset* shows the evolution of the FWHM as a function of current for a modulation p-doped QD-only SLD, which is processed by post-growth intermixing

Figure 5 illustrates the evolution of the FWHM with increasing injection current. Under low injection currents, the GS makes the main contribution to the emission. As the current is increased, the emission spectra are broadened to the short wavelength side, which is attributed to the filling of the QD’s ES1. Due to simultaneous contribution from the QD’s GS and ES1, the emission bandwidth is increased from 83 nm (at 150 mA) to 130 nm (at 300 mA) (see Fig. 4). This is in line with our previous work on modulation p-doped QD-only SLDs [33], demonstrated in the inset in Fig. 5, where the introduction of the excited state emission increases the spectral bandwidth. However, for this QD-only SLD, as the current is further increased, the spectrum becomes dominated by the ES leading to a *reduction* in FWHM to a value similar to that obtained for the GS. These effects have also been observed by other groups [34–36]. Here, for the hybrid QW/QD SLD, the QD emission saturates and the emission due to the lowest energy transition of the QW gradually increases with increasing current. Due to the combined contribution from both the GS and the ES1 of the QDs and the lowest energy transition of the QW, the device exhibits an emission with a 3-dB bandwidth of ~210 nm at 600 mA. Further increasing the current induces higher energy transitions of the QW, and as a result, an emission spectrum with a 3-dB bandwidth of 289 nm, centered at 1200 nm, is achieved with a corresponding output power of 2.4 mW.

**Fig. 6:**
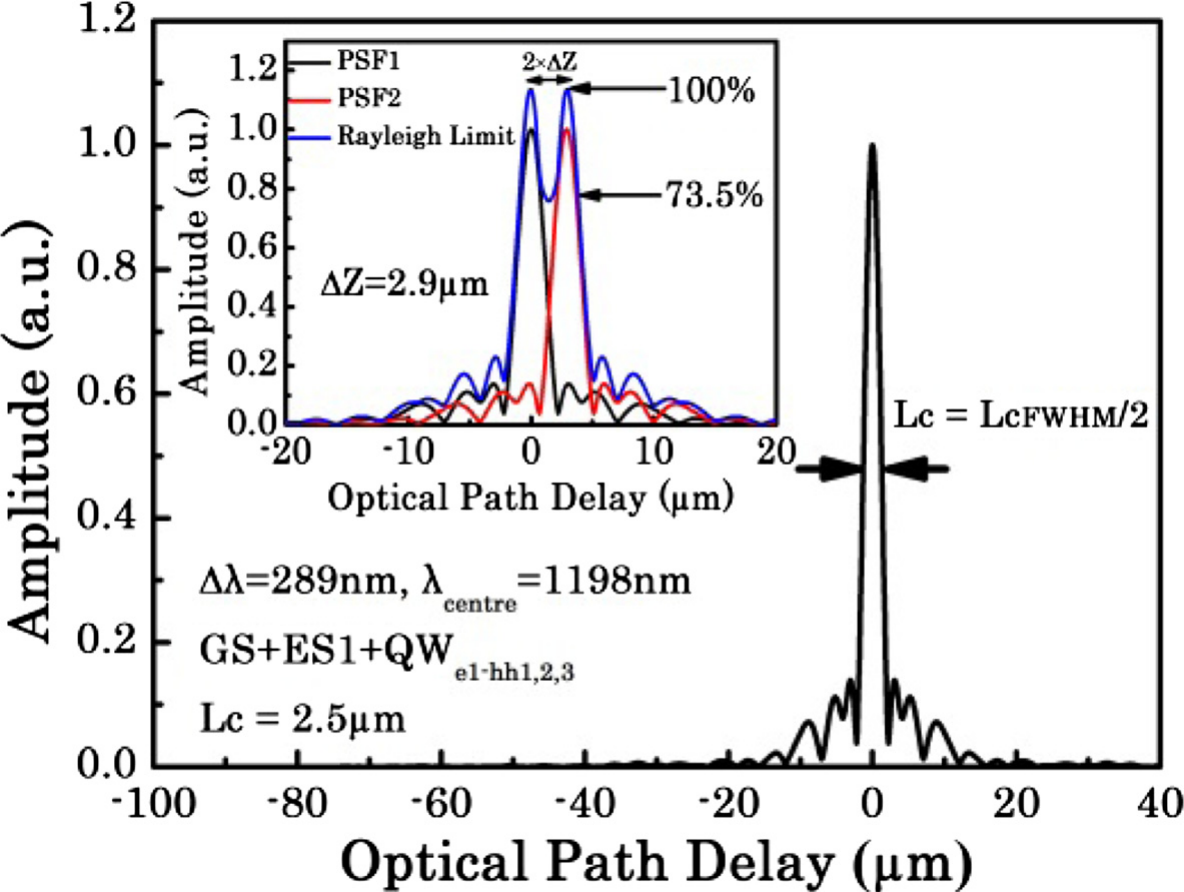
Self-coherence function calculated from the optimal spectrum (FWHM = 289 nm). The self-coherence function is obtained by the inverse Fourier transform of the power spectral density (PSD). The *inset* shows the plot when the two PSF functions satisfy the Rayleigh criterion

In the following, the device is assessed for application in OCT imaging systems. Figure 6 plots the self-coherence function, which is given by the inverse Fourier transform of the power spectral density and can be regarded as the point spread function (PSF) of the imaging system [2]. If we assume the emission spectrum to be Gaussian, the coherence length (and hence the axial resolution) would be half of the FWHM of the self-coherence function. For the optimized emission spectrum obtained from the hybrid QW/QD SLD where the 3-dB bandwidth of 289 nm is centered at 1200 nm, a resolution of 2.5 μm is thus predicted. In order to accommodate the undesirable side lobe generated by the more complex spectral structure, we need to introduce the Rayleigh criterion for resolution, which is explored in the inset in Fig. 6. Here, a resolution of 2.9 μm is obtained, indicating that a 0.4-μm penalty is introduced to the OCT system axial resolution due to the PSF side lobes (due to the non-Gaussian emission spectrum) of the device [2, 37]; this “penalty” may be reduced by reducing the spectral dips between QD GS and ES, and QD ES and QW. This can be achieved by chirping the QDs to have different emission wavelengths [38] or post-growth intermixing [39].

## Conclusions

We have observed significant emission from higher order QW transitions (e1-hh2, e1-hh3) in hybrid QW/QD structures and have attributed this to either the direct strain modulation of the QW and/or the creation of a piezoelectric potential due to the strained QDs. We have exploited this finding in the QW/QD SLD, which exhibits a spectral bandwidth of 290 nm with a corresponding output power of 2.4 mW. Assessment of this device for optical coherence tomography indicates that an axial resolution of 2.9 μm should be possible.

## Abbreviations

DWELL: dot-in-well
EL: electron luminescence
FWHM: full width at half maximum
L-I: light-current-voltage
MBE: molecular beam epitaxy
OCT: optical coherence tomography
PC: photocurrent
QD: quantum dot
QW: quantum well
SLD: superluminescent light-emitting diode

## References

[1] Huang D, Swanson EA, Lin CP, Schuman JS, Stinson WG, Chang W (1991). Optical coherence tomography. Science.

[2] Ackay C, Parrein P, Rolland JP (2002). Estimation of longitudinal resolution in optical coherence imaging. Appl Opt.

[3] Salomatina E, Yaroslavsky AN (2008). Evaluation of the in vivo and ex vivo optical properties in a mouse ear model. Phys Med Biol.

[4] Ebiko Y, Muto S, Suzuki D, Itoh S, Shiramine K, Haga T (1998). Island size scaling in InAs/GaAs self-assembled quantum dots. Phys Rev Lett.

[5] Raymond S, Fafard S, Poole PJ, Wojs A, Hawrylak P, Charbonneau S (1996). State filling and time-resolved photoluminescence of excited states in InxGa12xAs/GaAs self-assembled quantum dots. Phys Rev B.

[6] Greenwood PDL, Childs DTD, Groom KM, Stevens BJ, Hopkinson M, Hogg RA (2009). Tuning superluminescent diode characteristics for optical coherence tomography systems by utilizing a multicontact device incorporating wavelength-modulated quantum dots. IEEE J Sel Top Quant Electron.

[7] Zhang ZY, Hogg RA, Lv XQ, Wang ZG (2010). Self-assembled quantum dot superluminescent light-emitting diodes. Adv Opt Photon.

[8] Chen S, Tang M, Jiang Q, Wu J, Dorogan VG, Benamara M (2014). InAs/GaAs quantum-dot superluminescent light-emitting diode monolithically grown on a Si substrate. ACS Photonics.

[9] Tsuda M, Inoue T, Kita T, Wada O (2010). Broadband light sources using InAs quantum dots with InGaAs strained-reducing layers. Phys Status Solidi C.

[10] Chen S, Tang M, Wu J, Jiang Q, Dorogan V, Benamara M (2015). Long-wavelength InAs/GaAs quantum-dot light emitting sources monolithically grown on Si substrate. Photonics.

[11] Krstajic N, Smith LE, Matcher SJ, Childs DTD, Bonesi M, Greenwood PDL (2010). Quantum dot superluminescent diodes for optical coherence tomography: skin imaging. IEEE J Sel Top Quant Electron.

[12] Greenwood PDL, Childs DTD, Kennedy K, Groom KM, Hugues M, Hopkinson M (2010). Quantum dot superluminescent diodes for optical coherence tomography: device engineering. IEEE J Sel Top Quant Electron.

[13] Chen S, Zhou K, Zhang Z, Childs DTD, Hugues M, Ramsay A (2012). Ultra-broad spontaneous emission and modal gain spectrum from a hybrid quantum well/quantum dot laser structure. Appl Phys Lett.

[14] Chen S, Zhou K, Zhang Z, Orchard JR, Childs DTD, Hugues M (2013). Hybrid quantum well/quantum dot structure for broad spectral bandwidth emitters. IEEE J Sel Top Quant Electron.

[15] Chen S, Zhou K, Zhang Z, Wada O, Childs DTD, Hugues M (2012). Room temperature simultaneous three-state lasing in hybrid quantum well/quantum dot laser. Electron Lett.

[16] Davies JH (1999). Quantum dots induced by strain from buried and surface stressors. Appl Phys Lett.

[17] Tsatsul’nikov AF, Egorov AY, Zhukov AE, Kovsh AR, Ustinov VM, Ledentsov NN (1997). Modulation of a quantum well potential by a quantum-dot array. Semiconductor.

[18] Drexler W, Morgner U, Ghanta RK, Kartner FX, Schuman JS, Fujimoto JG (2001). Ultrahigh-resolution ophthalmic optical coherence tomography. Nat Med.

[19] Liu HY, Sellers IR, Badcock TJ, Mowbray DJ, Skolnick MS, Groom KM (2004). Improved performance of 1.3 μm multilayer InAs quantum-dot lasers using a high-growth-temperature GaAs spacer layer. Appl Phys Lett.

[20] Chen S, Tang M, Wu J, Jiang Q, Dorogan VG, Benamara M (2014). 1.3 μm InAs/GaAs quantum-dot laser monolithically grown on Si substrates operating over 100°C. Electron Lett.

[21] Bhattacharya P, Ghosh S (2002). Tunnel injection In0.4Ga0.6As/GaAs quantum dot lasers with 15 GHz modulation bandwidth at room temperature. Appl Phys Lett.

[22] Zeimer U, Bugge F, Gramlich S, Smirnitski V, Weyers M, Tränkle G (2001). Evidence for strain-induced lateral carrier confinement in InGaAs quantum wells by low-temperature near-field spectroscopy. Appl Phys Lett.

[23] Kash K, Mahoney DD, Van der Gaag BP, Gozdz AS, Harbison JP, Florez LT (1992). Observation of quantum dot levels produced by strain modulation of GaAs–AlGaAs quantum wells. J Vac Sci Technol B.

[24] Sopanen M, Lipsanen H, Ahopelto J (1995). Strain-induced quantum dots by self-organized stressors. Appl Phys Lett.

[25] NextNano GmbH. 2012. Retrieved from http://www.nextnano3/index.htm.

[26] Semenov T, Shidlovski VR, Safin SA (1993). Wide spectrum single quantum well superluminescent diodes at 0.8 μm with bent optical waveguide. Electron Lett.

[27] Fry PW, Itskevich IE, Mowbray DJ, Skolnick MS, Finley JJ, Barker JA (2000). Inverted electron–hole alignment in InAs-GaAs self-assembled QD. Phys Rev Lett.

[28] Miller DAB, Chemla DS, Damen TC, Gossard AC, Wiegmann W, Wood TH (1984). Band-edge electroabsorption in QW structure: the quantum-confined Stark effect. Phys Rev Lett.

[29] Skolnick MS, Tapster PR, Bass SJ, Pitt AD, Apsley N, Aldred SP (1986). Investigation of InGaAs-InP quantum wells by optical spectroscopy. Semicond Sci Technol.

[30] Marko IP, Adams AR, Sweeney SJ, Mowbray DJ, Skolnick MS, Liu HY (2005). Recombination and loss mechanisms in low-threshold InAs/GaAs 1.3-μm quantum-dot lasers. IEEE J Sel Top Quant Electron.

[31] Shchekin O, Deppe D (2002). 1.3 μm InAs quantum dot laser with T0 = 161 K from 0 to 80 °C. Appl Phys Lett.

[32] Li X, Jin P, An Q, Wang Z, Lv X, Wei H (2012). Improved continuous-wave performance of two-section quantum-dot superluminescent diodes by using epi-down mounting process. IEEE Photon Technol Lett.

[33] Zhang ZY, Jiang Q, Luxmoore IJ, Hogg RA (2009). A p-type-doped quantum dot superluminescent LED with broadband and flat-topped emission spectra obtained by post-growth intermixing under a GaAs proximity cap. Nanotechnology.

[34] Xin Y, Martinez A, Nilsen TA, Moscho A, Li Y, Gray AL (2007). 1.3 μm quantum dot multi-section superluminescent diode with extremely broad bandwidth (>150 nm). IEEE Photon Technol Lett.

[35] Rossetti M, Markus A, Fiore A, Occhi L, Vele C (2005). Quantum dot superluminescent diodes emitting at 1.3 μm. IEEE Photon Technol Lett.

[36] Wang ZC, Jin P, Lv XQ, Li XK, Wang ZG (2011). High-power quantum dot superluminescent diode with integrated optical amplifier section. Electron Lett.

[37] Zhou KJ, Jiang Q, Zhang ZY, Chen SM, Liu HY, Lu ZH (2012). Quantum dot selective area intermixing for broadband light sources. Opt Express.

[38] Ray SK, Groom KM, Beattie MD, Liu HY, Hopkinson M, Hogg RA (2006). Broad-band superluminescent light-emitting diodes incorporating quantum dots in compositionally modulated quantum wells. IEEE Photon Technol Lett.

[39] Zhang ZY, Jiang Q, Hopkinson M, Hogg RA (2010). Effects of intermixing on modulation p-doped quantum dot superluminescent light emitting diodes. Opt Express.

